# A comparison of sequential density ultracentrifugation and density gradient ultracentrifugation for the proteomic analysis of high-density lipoprotein

**DOI:** 10.3389/fcvm.2026.1817349

**Published:** 2026-05-15

**Authors:** Jack D. Beazer, Stefan Ljunggren, Helen Karlsson, Ian G. Davies, Dilys J. Freeman

**Affiliations:** 1 School of Cardiovascular and Metabolic Health, Glasgow Cardiovascular Research Centre, University of Glasgow, Glasgow, United Kingdom; 2 Occupational and Environmental Medicine Center in Linköping, and Department of Health, Medicine and Caring Sciences, Linköping University, Linköping, Sweden; 3 Research Institute of Sport and Exercise Science, Liverpool John Moores University, Liverpool, United Kingdom

**Keywords:** apolipoproteins, cholesterol, iodixanol, lipoproteins, ultracentrifugation, vascular biology

## Abstract

**Introduction:**

With the failure of high-density lipoprotein (HDL) cholesterol raising therapies to reduce cardiovascular risk, attention has turned towards HDL composition and function. There are several techniques for the isolation of lipoproteins from plasma, with density ultracentrifugation considered the gold-standard method. It is not known which form of density ultracentrifugation is optimal for HDL composition and function analyses.

**Methods:**

This study compared HDL composition by nLC-MS/MS and ELISA, subclass distribution by gel electrophoresis, and *in vitro* vascular anti-inflammatory function in HDL fractions isolated by sodium bromide sequential density ultracentrifugation (SDU) and iodixanol density gradient ultracentrifugation (DGU).

**Results:**

HDL composition differed between the two isolation techniques, with DGU-isolated HDL fractions containing a higher total protein content than SDU (10.14 ± 1.49 mg/mL compared to 3.18 ± 1.10 mg/mL respectively, *p* < 0.001, mean ± SD) but lower proteomic detection of key HDL proteins such as apolipoprotein A-I, apolipoprotein A-II and paraoxonase-1 in DGU-isolated HDL. HDL subclass distribution could not be determined in DGU HDL fractions due to contaminating plasma proteins. Vascular anti-inflammatory function was higher in DGU HDL (77.5 ± 4.4 % compared to 58.3 ± 4.8%, mean ± SD, *p* = 0.014) possibly due to the presence of confounding plasma proteins.

**Conclusion:**

Further steps may need to be added to DGU methods to obtain a ‘cleaner’ HDL fraction and therefore currently SDU is better suited to studies of HDL composition and function.

## Introduction

High-density lipoprotein cholesterol (HDL-C) was first established as an inverse predictor of cardiovascular disease (CVD) risk in the Framingham heart study in the 1970s and has since received continuous attention for over 50 years (1, 2). HDL provides vascular protection through reverse cholesterol transport, and it was thought that increasing HDL-C would reduce cardiovascular risk. Despite being successful at increasing HDL-C, pharmacological interventions did not modify cardiovascular risk and as such pharmacological modulation of HDL-C was abandoned (3–5). Furthermore, recent epidemiological and genetic evidence has failed to link gene variants associated with HDL concentration to cardiovascular outcomes or to the clinical manifestation of CVD (6–8). Attention has now turned towards HDL composition and function and their effects on vascular protection. Measurement of these metrics necessitates the isolation of the lipoprotein. It is therefore important to understand the impact of the isolation method employed on HDL's composition and function.

HDL was shown in 2005 to have a proteome distinct from other lipoproteins (9). As of 2022 the HDL proteome watch deemed 251 proteins to be HDL-associated, based on their identification in at least three separate studies of HDL protein composition. These are involved in a range of functions such as lipid transport, coagulation, cell-binding and immunity (10). Apolipoprotein A-I (apoA-I) makes up ∼70% of HDL protein content. ApoA-I is the primary functional protein that facilitates cholesterol efflux and vascular protective HDL properties (such as anti-inflammatory and antioxidant) through interaction with membrane receptors and transporters such as ABCA1. Apolipoprotein A-II (apoA-II) accounts for ∼20% of HDL protein and is an important modulator of HDL size and efflux capacity (11, 12). HDL also hosts other apolipoproteins (apoC, D, E, M, F), though these are not obligate HDL apolipoproteins but are recruited through interaction with triglyceride-rich lipoproteins. Other proteins on HDL include paraoxonase-1, an antioxidant enzyme that can prevent oxidation of low-density lipoprotein, a key step in atheroma formation (13, 14).

The gold standard technique for the isolation of HDL from plasma is density ultracentrifugation (15). There are two predominant methods of density ultracentrifugation: sequential density ultracentrifugation (SDU) and density gradient ultracentrifugation (DGU). SDU includes multiple centrifugation steps, the first of which removes very low- and low-density lipoproteins (VLDL/LDL) before a second step which isolates the HDL fraction. The density media used in SDU are typically salt-based and use either sodium or potassium bromide, though salt is hyperosmotic and can cause loss of water in the samples, thereby increasing the density of lipoproteins. There is evidence of salt-induced alterations in HDL-associated apolipoprotein content, such as apolipoprotein E (16). In contrast, DGU is a single step process which separates all the lipoprotein classes at once, due to the ability of the density solution to form a density gradient increasing from the top to the bottom of the ultracentrifuge tube. The density solutions used are typically biologically inert substances such as iodixanol or sucrose. Iodixanol is non-osmotic and non-hygroscopic and therefore maintains lipoprotein density and integrity (17), while sucrose is hygroscopic and may impact hydration (18). After centrifugation, the whole ultracentrifuge tube is fractionated, and the lipoprotein content identified by density and/or apolipoprotein content. Other techniques for HDL isolation include immunoaffinity and size exclusion chromatography, though these are less extensively employed and are readily contaminated with plasma protein aggregates or other lipoproteins (15).

Previous studies have compared the effect of HDL isolation methods more broadly on HDL protein composition and found differences between ultracentrifugation, immunoaffinity and precipitation (19), or have compared SDU and DGU for studies of HDL anti-inflammatory function (20). However, there is limited literature comparing HDL protein composition measured by proteomics using different ultracentrifugation techniques. This study therefore aimed to address this gap by comparing the isolation of HDL by SDU using NaBr density buffers and DGU using iodixanol solution, for their effects on the subsequent measurement of HDL composition, primarily proteomic assessment of isolated HDL, and HDL *in vitro* anti-inflammatory function.

## Materials and methods

### Study samples

Plasma samples were archival from a previously performed study of muscle triglyceride synthesis (21). The study was carried out in accordance with the World Medical Association Declaration of Helsinki and all participants provided written informed consent. The study was conducted with ethical approval from the West of Scotland Research Ethics Committee 4 (16/WS/0002). Samples from five healthy control men and five men with impaired glucose regulation [IGR, defined as a HbA1c between 6.0 and 6.4% (43.8–47.4 mmol/mol) per the National Institute for Health and Care Excellence NG28 and World Health Organisation guidance] were selected at random (baseline characteristics detailed in Supplementary Table 1). Samples from both groups were included due to sample availability and to ensure the methodological comparison was conducted across the full spectrum of HDL composition and functional biology given the known differences between healthy control and IGR HDL (22). Blood was drawn from the median cubital vein into tubes containing EDTA and plasma isolated from whole blood by centrifugation at 1,500 × *g* at 4 °C for 15 min. Aliquoted plasma was stored at −80 °C until use. Each sample was isolated using both SDU and DGU for a final *n* of 10 per isolation method.

### Isolation of HDL

Sodium bromide SDU was performed as previously described (23). Density solutions were prepared as follows: 1.006 g/mL (11.4 g NaCl, 0.1 g EDTA, and 1 mL 1 M NaOH made to 1 L in dH_2_O), 1.182 g/mL (29.98 g NaBr in100 mL 1.006 g/mL solution), 1.063 g/mL (1.006 g/mL and 1.182 g/mL solutions in a 2:1 ratio), 1.478 g/mL (78.32 g NaBr in 100 mL 1.006 g/mL solution) and 1.21 g/mL (1.063 g/mL and 1.478 g/mL solutions in a 2:1 ratio). All solutions were checked before use with a density meter (DMA35 V3, Anton Paar, St Albans, Hertfordshire, UK), and adjusted if necessary, with further NaBr or the 1.006 g/mL solution. VLDL and LDL were first isolated from plasma by flotation at a density of 1.063 g/mL. Plasma (500 μL) was combined with 250 μL 1.182 g/mL NaBr solution and overlayed with 250 μL 1.063 g/mL NaBr solution. The samples were then centrifuged in a Beckman Coulter TLA 120.2 rotor and Optima MAX-TL centrifuge at 100,000 RPM (355,040 × *g* average) for 2.5 h at 23 °C. After this time, 500 µL supernatant (containing VLDL/LDL) was removed. Next HDL was isolated at a density of 1.21 g/mL. The remaining infranatant from the previous ultracentrifugation was combined with 250 µL of 1.478 g/mL NaBr solution and overlayed with 250 µL 1.21 g/mL solution. Samples were centrifuged again at 100,000 RPM (355,040 × g average) for 5 h at 23 °C. The resulting top 500 µL supernatant contained HDL. HDL fractions were desalted using PD MiniTrap G-25 columns (Cytiva, Little Chalfont, Buckinghamshire, UK, #28918007) according to the manufacturer's protocol. Desalted HDL fractions were stored at −80 °C until use.

**Figure 1:**
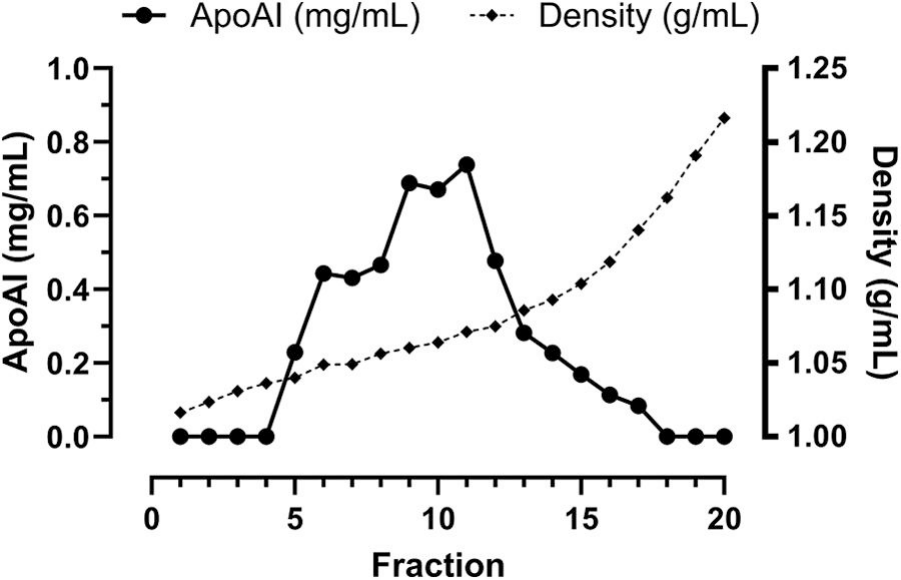
The apoA-I content and density of each 500 μL fraction resulting from density gradient ultracentrifugation with iodixanol. Each 500 µL fraction from a blank ultracentrifuge tube containing only iodixanol solutions had its density measured by refractometer (diamonds and dashed line). Each fraction from one control sample was assessed for apoA-I content by ELISA (black circles and solid lines). Fractions 6-13 were deemed to contain HDL. For the remaining samples, these fractions were pooled and concentrated back to the original plasma volume before further analysis.

Iodixanol DGU was performed as previously described (24). Iodixanol solutions (15% w/v and 24% w/v) were prepared by diluting Optiprep (Sigma-Aldrich, Gillingham, Dorset UK, #D1556) with Dulbecco's modified phosphate buffered saline with 0.9% NaCl. Participant plasma was mixed with Optiprep to a final concentration of 18.5% (w/v) iodixanol to form the working sample. Initially, 4 mL of the 15% (w/v) iodixanol solution was loaded into polypropylene centrifuge tubes (16 × 70 mm, 11.2 mL OptiSeal, Beckman Coulter, #362181). The working sample (3 mL) was under-layered below the 15% iodixanol layer. Finally, the 24% iodixanol solution (4 mL) was under-layered into the bottom of the centrifuge tube. Loaded centrifuge tubes were sealed with OptiSeal plugs and housed in a Beckman Coulter NVT65 rotor (Beckman Coulter, Brea, California, USA). The samples were centrifuged in a Beckman Coulter XPN ultracentrifuge (Beckman Coulter, Brea, California, USA) at 65,000 RPM (352,534 × *g* average) for 3 h and 10 min at 16 °C. After centrifugation samples were fractionated into twenty 500 µL volumes with a Labonco Autodensiflow (Labonco, Kansas City, MO, USA), Watson-Marlow 101U/R pump (Falmouth, Cornwall, United Kingdom) and Gilson FC203P fraction collector (Gilson Inc., Middleton, WI, USA). The density of each fraction was measured using an Abbe refractometer (Bellingham and Stanley, Basingstoke, UK) and the refractive index converted to density using the following equation: *ρ* = *η*a—b (where a = 3.4193, b = 3.56, *η* = refractive index, and *ρ* = density). Fractions 6–13 containing apoA-I were pooled together and concentrated back to the initial volume of plasma using Vivaspin 6 centrifugal concentrators (Cytiva # 28932296) with a 10,000 Da molecular weight cut off (Figure 1). HDL was isolated by DGU at a density of between 1.053 g/mL and 1.093 g/mL.

### Measurement of HDL composition

HDL apoA-I and serum amyloid A1 (SAA-1) content were measured using enzyme linked immunosorbent assay (ELISA) kits (#DY3664 and #DY3019, R&D Systems, Abingdon, United Kingdom) according to the manufacturer's instructions and using the appropriate ancillary kits. HDL total protein content was measured by the Bradford assay (25). HDL total cholesterol content was measured using a colorimetric cholesterol quantitation kit (Sigma-Aldrich, #MAK043) according to the manufacturer's protocol.

### Measurement of HDL subclass distribution

HDL subclass distribution was measured using native PAGE as previously described (22). Briefly, NativeMark unstained protein standard (5 µL, Thermo Fisher Scientific #LC0725) was electrophoresed alongside HDL samples. Gels were stained for protein in 50 mL QC Colloidal Coomassie stain (Bio-Rad Laboratories #1610803) and imaged using a LI-COR Odyssey FC scanner. The molecular weights of each protein in the standard were converted to diameter (nanometres) using the calculator found at ‘https://www.nanocomposix.com/pages/molecular-weight-to-size-calculator’, based on mathematical modelling described elsewhere (26). Standardised retention factors were calculated for each marker band and a linear standard curve drawn. This standard curve was used to calculate the maximum and minimum RF values for each of the following HDL subclasses based on their diameter: 2b 9.7–12.9 nm, 2a 8.8–9.7 nm, 3a 8.2–8.8 nm, 3b 7.8–8.2 nm, 3c 7.2–7.8 nm (27).

### Proteomic assessment of HDL composition

Proteomic analyses were performed according to (22) with some modifications. HDL (10 μg proteins) was reduced by adding 2 µL of 25 mM dithiothreitol and incubating for 1 h at 60 °C, alkylated by adding 2 µL of 75 mM iodoacetamide and incubated for 15 min at room temperature before digestion with trypsin (1:25) overnight at 37 °C. The resulting peptides were desalted using C18 ZipTip (Merck Millipore), lyophilized and reconstituted in 0.1% formic acid. Peptide concentrations were determined using a Nanodrop 1000 system (Thermo Fisher Scientific).

Peptides (200 ng) were separated on a 20 cm EASY-Spray C18 connected to an EASY-nLC 1200 (Thermo Scientific, Waltham, MA, USA) using a linear gradient of 0.1% formic acid in water (A) and 0.1% formic acid in 80% acetonitrile (B) (7%–40% B over 75 min followed by 40%–100% B over 13 min and 2 min of holding at 100% B). Automated online analyses were performed with a QExactive HF mass spectrometer (Thermo Scientific) with a nano-electrospray source and a top10 data-dependant method. Raw files were searched using MaxQuant v.2.0.2.0 (Max Planck Institute of Biochemistry, Martinsried, Germany) against a Uniprot Human database (downloaded 9th February 2022) with the following parameters: trypsin was used as digestion enzyme; maximum number of missed cleavages 2; fragment ion mass tolerance 0.50 Da; parent ion mass tolerance 5.0 ppm; fixed modification—carbamidomethylation of cysteine; variable modifications—N-terminal acetylation and methionine oxidation Proteins with at least two peptides of which one was unique and identified in at least 50% of the HDL samples in a given isolation method were included in further analysis. Label-free quantification (LFQ) was performed using the built-in LFQ algorithm.

### HDL *in vitro* anti-inflammatory function

HDL *in vitro* anti-inflammatory function was measured as previously described (22). Briefly, human microvascular endothelial cells (HMEC-1, ATCC-CRL-3243, LGC Standards, Middlesex, UK) were cultured according to manufacturer recommendations. HMEC-1 were preincubated with HDL (based on 300 µg/mL apoA-I) for four hours before the addition of 5 ng/mL TNF*α* for 24 h. Cells were lysed in radioimmunoprecipitation assay buffer (150 mM NaCl, 50 mM Tris-HCl pH 7.4, 1% v/v Triton X-100, 0.5% m/v sodium deoxycholate, 0.1% m/v sodium dodecyl sulphate, 1 mM ethylenediaminetetraacetic acid, 10 mM NaF, pH 7.4, supplemented with 1 × Roche Complete mini protease inhibitor cocktail tablet (Sigma-Aldrich #11836153001)) and western blotting performed to detect vascular cell adhesion molecule 1 (VCAM-1), using a mouse anti-human VCAM-1 antibody (Santa Cruz Biotechnology, sc-13160) and donkey anti-mouse IgG IRDye 800cw antibody (LI-COR, # 926-32212). Total protein normalisation was used as a loading control and blots were visualised using a LI-COR Odyssey FC scanner.

### Statistical analysis

All data was analysed using mixed effects models using Minitab (State College, PA, USA) software version 20.3, ensuring normal distribution of residuals, followed by *post hoc* Tukey test. Non-normal residual distributions were rectified by log transformation of the input data. For all analyses, statistical significance was assumed at *p* < 0.05. Graphs were drawn with GraphPad (Boston, MA, USA) Prism version 10.3.1. For the generation of the heatmap of proteomic data, the non-parametric LFQ intensities were standardised using *z*-scores with the median and median absolute deviation in place of mean and standard deviation. Bland-Altman analysis was by plotting the difference in measured value between paired samples against the average measured value. The mean bias was calculated as the average difference between measured values. The limits of agreement were calculated as ± 1.96 standard deviations from the mean difference as per (28). As HDL carries multiple apoA-I molecules and HDL-C varies between individuals, dosing by apoA-I may mean that cells are exposed to different HDL and protein concentrations. To account for this, the analysis of the anti-inflammatory assay included HDL-C as a covariate.

## Results

### HDL apoA-I, total cholesterol and total protein content after SDU and DGU

**Figure 2:**
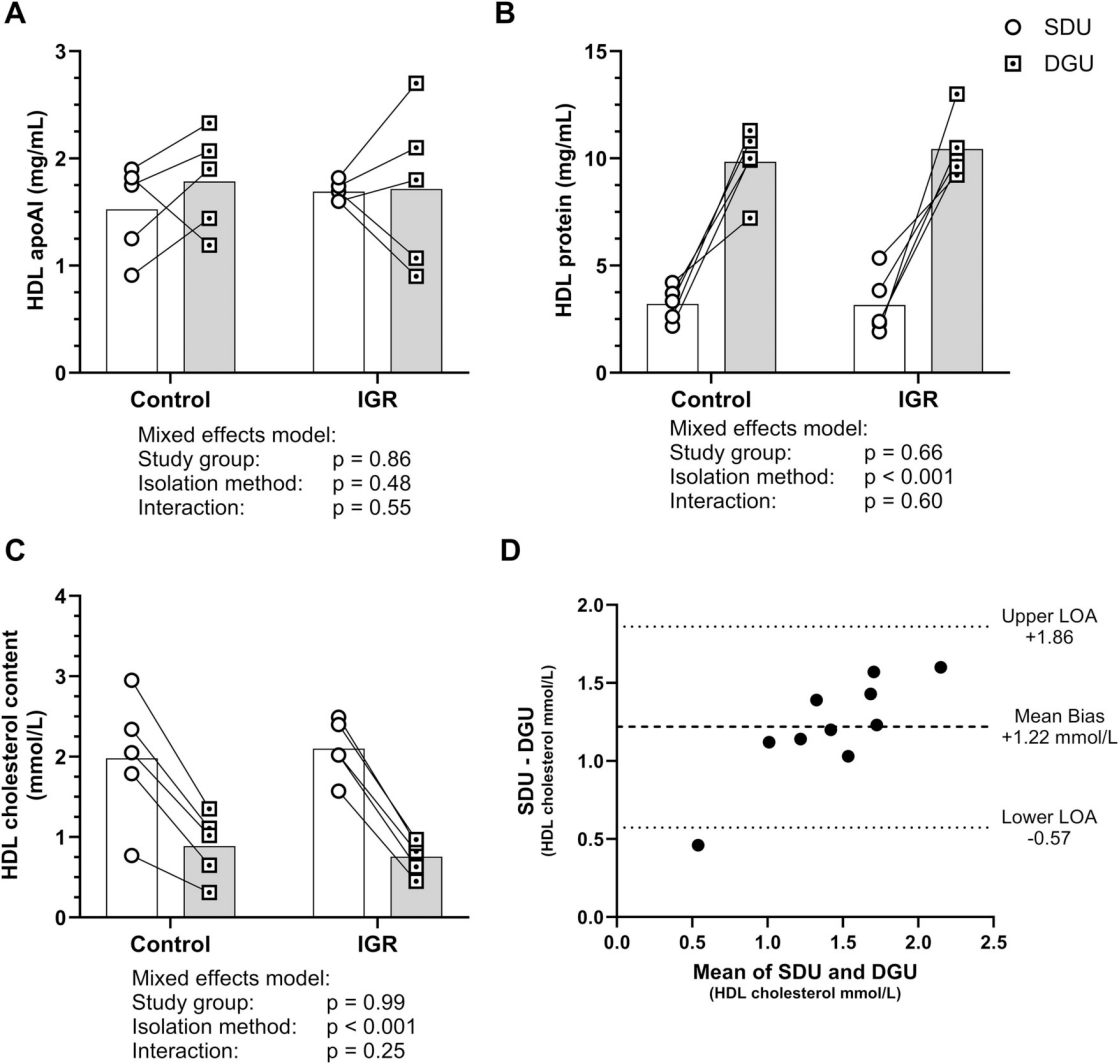
HDL components after SDU and DGU. **(A)** HDL apoA-I content, **(B)** HDL total protein content and **(C)** HDL total cholesterol content. Circles indicate SDU and dotted squares indicate DGU. Mean represented by bars and paired samples connected by line. Data analysed by mixed effects model and statistical significance assumed at *p* < 0.05. **(D)** Bland-Altman analysis of HDL cholesterol content after SDU and DGU. Mean bias between the isolation methods was 1.22 mmol/L and the limits of agreement were between 0.57 mmol/L and 1.86 mmol/L. IGR, impaired glucose regulation; SDU, sequential density ultracentrifugation; DGU, density gradient ultracentrifugation; LOA, limit of agreement. *N* = 10 SDU and *N* = 10 DGU.

HDL apoA-I content did not differ by HDL isolation technique (Figure 2A). However, HDL protein and cholesterol content were significantly higher and lower respectively after DGU compared to SDU (10.14 ± 1.49 mg/mL compared to 3.18 ± 1.10 mg/mL, *p* < 0.001, mean ± SD, Figure 2B, 0.82 ± 0.32 mmol/L compared to 2.04 ± 0.59 mmol/L, *p* < 0.001, mean ± SD, Figure 2C). A Bland- Altman plot for HDL cholesterol content (Figure 2D) showed the mean bias ± SD between SDU and DGU was 1.22 ± 0.33 mmol/L and the limits of agreement were between 0.57 mmol/L and 1.86 mmol/L, with larger differences between the methods with higher HDL cholesterol content.

### HDL subclass distribution after SDU and DGU

**Figure 3:**
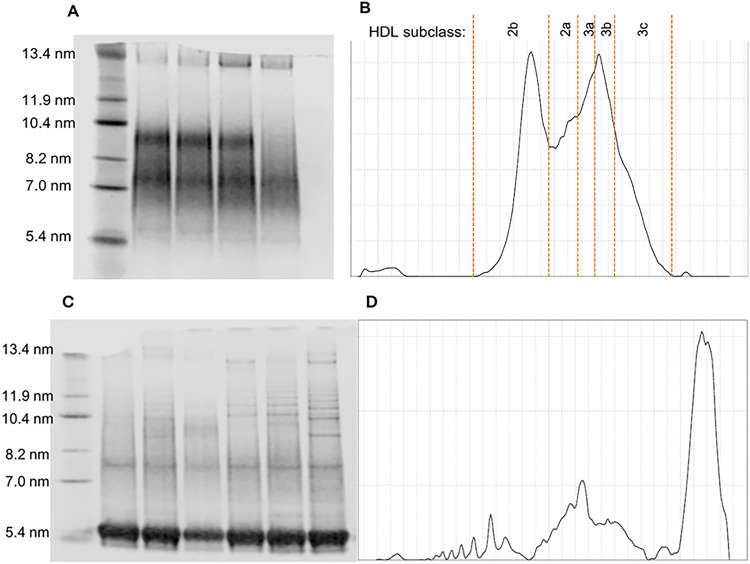
HDL subclass distribution after isolation by SDU and DGU. **(A)** Coomassie blue stained electrophoresed SDU isolated HDL with protein marker diameters indicated. **(B)** Representative profile of lane 2 of A with peaks corresponding to each HDL subclass delineated. **(C)** Coomassie blue stained electrophoresed DGU isolated HDL with protein marker diameters indicated. **(D)** Peak profile of the rightmost lane of the gel in C.

Electrophoresis of HDL isolated by SDU resulted in clean and interpretable lane profiles with distinct peaks corresponding to each subclass (Figures 3A,B). However, the electrophoresis of HDL isolated by DGU resulted in many smaller peaks which obscured the broader HDL subclasses and therefore could not be analysed (Figures 3C,D).

### HDL proteomic composition after SDU and DGU

**Figure 4:**
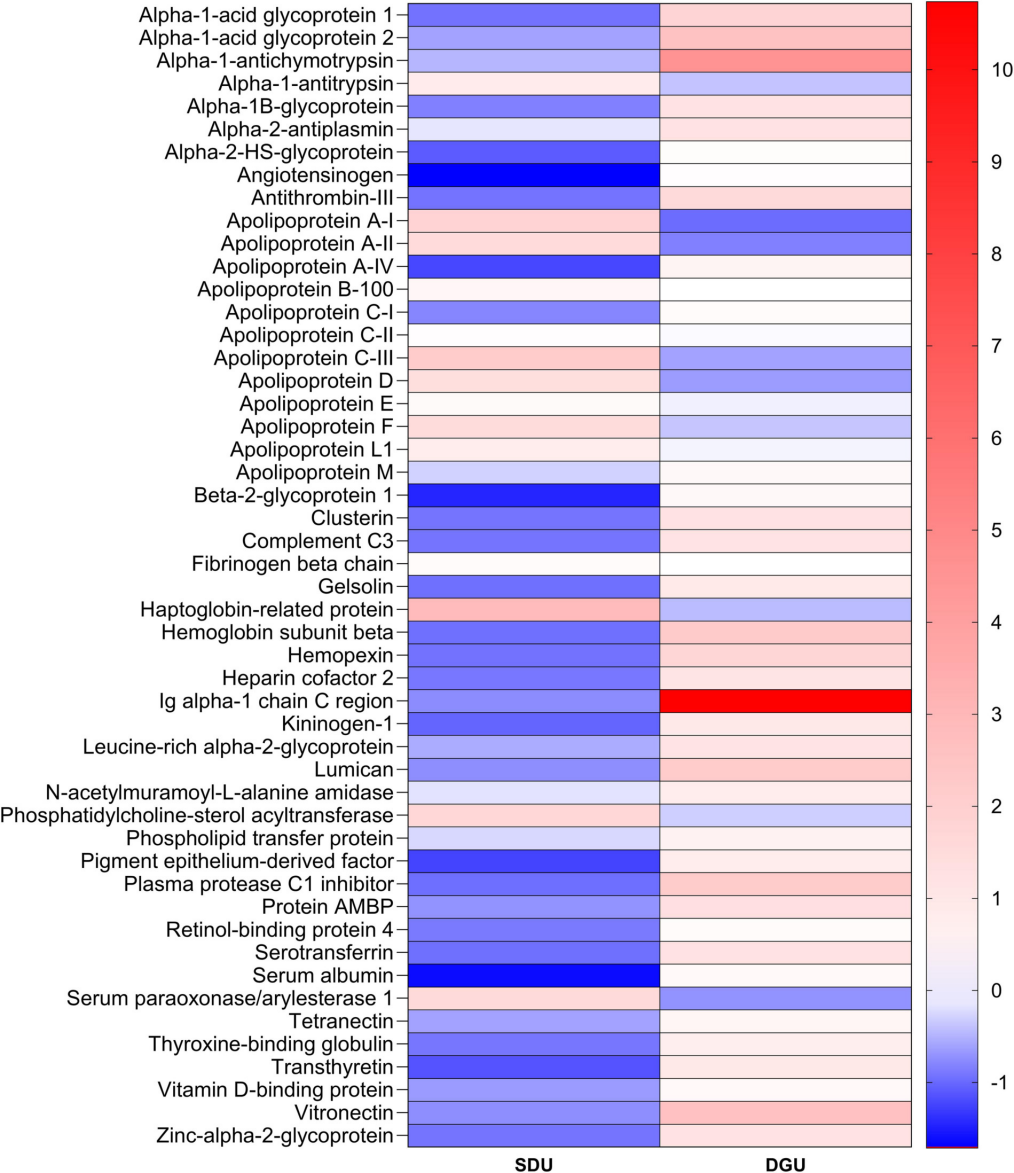
Heatmap of proteins identified on HDL isolated by both SDU and DGU. Heatmap plotted with the median absolute deviation adjusted LFQ intensity for each protein. SDU, sequential density ultracentrifugation; DGU, density gradient ultracentrifugation. *N* = 10 SDU and *N* = 10 DGU.

**Figure 5:**
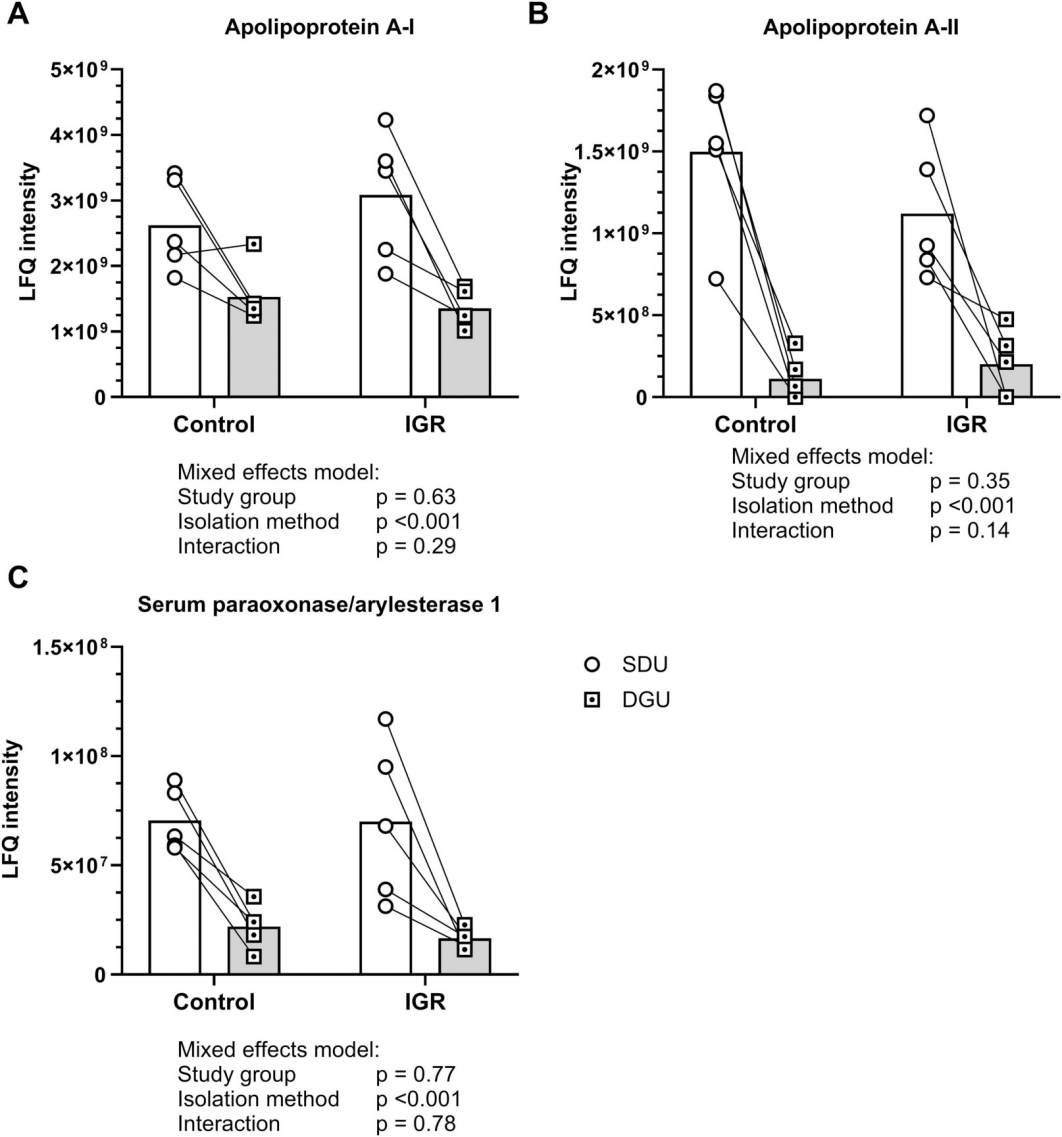
Abundance of selected HDL proteins identified on HDL from both naBr and iodixanol isolation techniques. Circles indicate SDU and dotted squares indicate DGU. Median represented by bars and paired samples connected by line. Data analysed by mixed effects model and statistical significance assumed at *p* < 0.05. IGR, impaired glucose regulation; SDU, sequential density ultracentrifugation; DGU, density gradient ultracentrifugation. *N* = 10 SDU and *N* = 10 DGU.

There were initial difficulties in preparing the DGU samples for proteomic analysis. After using the same peptide isolation and cleaning protocols as SDU, a broad peak centred around 244 nm, corresponding to iodixanol, was observed in the Nanodrop 1000 absorbance spectra of the DGU samples but not in the SDU samples (29). This was removed by five sequential buffer exchanges using 3 KDa MWCO centrifugal filters. Proteomic analysis of HDL isolated by both SDU and DGU revealed 97 HDL associated proteins. Of those, 50 were identified on HDL isolated by both SDU and DGU (Table 1), 5 were identified only on HDL isolated by SDU (including CETP and SAA-1, Table 2) and 42 were identified only on HDL isolated by DGU (Table 3). Full details of each identified protein can be found in the supplementary data file. The median standardised LFQ intensities of the proteins identified on HDL isolated by both ultracentrifugation techniques were plotted against each other in a heatmap (Figure 4). The heatmap revealed different abundances of identified proteins between the techniques, with a higher relative abundance of apolipoproteins on HDL isolated by SDU and a higher relative abundance of inflammatory and coagulation related proteins on HDL isolated by DGU. Of the proteins identified on HDL from both isolation techniques, the LFQ intensities of the key HDL proteins apoA-I, apoA-II and PON-1 were all significantly higher on HDL from SDU (Figure 5).

**Table 1:** Proteins identified on HDL after both SDU and DGU.

Alpha-1-acid glycoprotein 1	Gelsolin
Alpha-1-acid glycoprotein 2	Haptoglobin-related protein
Alpha-1-antichymotrypsin	Hemoglobin subunit beta
Alpha-1-antitrypsin	Hemopexin
Alpha-1B-glycoprotein	Heparin cofactor 2
Alpha-2-antiplasmin	Ig alpha-1 chain C region
Alpha-2-HS-glycoprotein	Kininogen-1
Angiotensinogen	Leucine-rich alpha-2-glycoprotein
Antithrombin-III	Lumican
Apolipoprotein A-I	N-acetylmuramoyl-L-alanine amidase
Apolipoprotein A-II	Phosphatidylcholine-sterol acyltransferase
Apolipoprotein A-IV	Phospholipid transfer protein
Apolipoprotein B-100	Pigment epithelium-derived factor
Apolipoprotein C-I	Plasma protease C1 inhibitor
Apolipoprotein C-II	Protein AMBP
Apolipoprotein C-III	Retinol-binding protein 4
Apolipoprotein D	Serotransferrin
Apolipoprotein E	Serum albumin
Apolipoprotein F	Serum paraoxonase/arylesterase 1
Apolipoprotein L1	Tetranectin
Apolipoprotein M	Thyroxine-binding globulin
Beta-2-glycoprotein 1	Transthyretin
Clusterin	Vitamin D-binding protein
Complement C3	Vitronectin
Fibrinogen beta chain	Zinc-alpha-2-glycoprotein

**Table 2:** Proteins only identified on HDL after SDU.

Cholesteryl ester transfer protein
Complement C4-A
Fibrinogen alpha chain
Ig lambda-1 chain C regions
Serum paraoxonase/lactonase 3

**Table 3:** Proteins only identified on HDL after DGU.

Actin, cytoplasmic 2	Ig kappa chain C region
Afamin Apolipoprotein(a)	Ig kappa chain V-II region RPMI 6410
Attractin	Ig kappa chain V-III region B6
Beta-Ala-His dipeptidase	Ig lambda-6 chain C region
Carboxypeptidase B2	Ig mu chain C region
Carboxypeptidase N subunit 2	Immunoglobulin lambda-like polypeptide 5
Ceruloplasmin	Insulin-like growth factor-binding protein complex acid labile subunit
Coagulation factor IX	Inter-alpha-trypsin inhibitor heavy chain H1
Coagulation factor V	Inter-alpha-trypsin inhibitor heavy chain H2
Coagulation factor X	Inter-alpha-trypsin inhibitor heavy chain H4
Complement C1r subcomponent	Kallistatin
Complement C1s subcomponent	Phosphatidylinositol-glycan-specific phospholipase D
Complement C4-B	Proteoglycan 4
Complement component C9	Prothrombin
Complement factor B	Serum amyloid P-component
Complement factor H	
Complement factor H-related protein 1	
Complement factor I	
Corticosteroid-binding globulin	
Fibrinogen gamma chain	
Haptoglobin	
Hemoglobin subunit alpha	
Ig gamma-1 chain C region	
Ig gamma-2 chain C region	
Ig gamma-3 chain C region	

### HDL *in vitro* anti-inflammatory function after SDU and DGU

**Figure 6:**
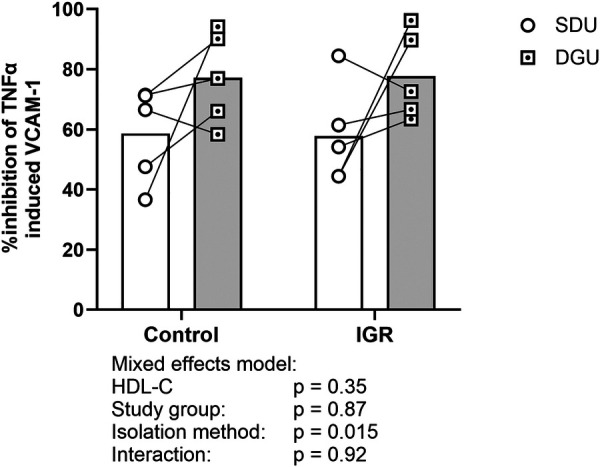
The effect of HDL isolation technique on HDL anti-inflammatory function. Data expressed as inhibition of TNF*α* induced VCAM-1 expression based on 300 µg/mL apoA-I. Circles indicate SDU and dotted squares indicate DGU. Mean represented by bars and paired samples connected by line. Data analysed by mixed effects model with HDL-C included as a covariate to account for individual differences in the distribution of apoA-I on HDL particles. Statistical significance was assumed at *p* < 0.05. IGR, impaired glucose regulation; SDU, sequential density ultracentrifugation; DGU, density gradient ultracentrifugation; HDL-C, high-density lipoprotein cholesterol; TNF*α*, tumour necrosis factor alpha; VCAM-1, vascular cell adhesion molecule 1. *N* = 10 SDU and *N* = 10 DGU.

HDL isolated by DGU had greater ability to inhibit TNF*α*-induced VCAM-1 expression in endothelial cells than HDL isolated by SDU (77.5 ± 4.4% compared to 58.3 ± 4.8% respectively, mean ± SD, *p* = 0.014, Figure 6).

## Discussion

This study explored how HDL isolated by SDU and DGU differed in terms of protein composition and anti-inflammatory function. HDL was isolated at a lower density in DGU, likely due to the lower osmotic forces on the lipoprotein particle compared to SDU (17). Direct assessment of HDL apoA-I concentration by ELISA showed that apoA-I levels did not differ between the isolation methods. However, a markedly higher HDL total protein content and lower HDL cholesterol content were observed in HDL isolated by DGU. This may be due to a greater recovery of HDL 3, or perhaps the different manner by which HDL is separated from plasma in each technique. In SDU, lipoproteins are floated to the top of the ultracentrifuge tube, with a density cushion between the floated lipoproteins and the remaining plasma sample; however, in DGU the whole plasma sample is separated throughout the entire ultracentrifuge tube across a density gradient and as such may lead to contamination of the HDL-containing fractions by plasma proteins. SDU centrifugation is performed for a longer duration than DGU (7 h 30 min total at 355,040 × g average compared to 3hrs 10 min at 352,534 × g average respectively) which may cause loosely associated HDL proteins to dissociate from HDL and be found in the lipoprotein depleted plasma (LPDP) fraction (16). Proteomic analysis of LPDP was not performed in this study but could have clarified whether this had occurred. Bland-Altman analysis suggested a methodological difference between SDU and DGU HDL cholesterol content, with DGU-isolated HDL consistently containing less cholesterol. The cholesterol quantitation assay in this study was an enzyme-coupled colorimetric assay; there is some evidence that iodixanol alters the performance of colorimetric and enzyme-based biochemical assays (30). As the iodixanol gradients were created at the same time using the same stock solutions, it is likely that the plasma samples contained a similar concentration of iodixanol which may have interfered with the assay to a similar degree in each sample, perhaps by quenching colour development or by impairing enzyme activity by obscuring substrate binding sites. This may also have had some impact on the protein measurements by Bradford assay.

It was not possible to compare the size distribution of HDL subfractions after isolation by the two different techniques as the lane profiles of DGU HDL were uninterpretable due to multiple bands forming on the gel. This was likely a consequence of the higher protein content of the HDL fraction after DGU given the gels were stained with the protein stain Coomassie blue G-250. It has been acknowledged that plasma proteins may interfere with protein-stained HDL sizing gels after DGU with iodixanol (24) in a previous study where SDU with potassium bromide was performed for the comparison of methods for assessing HDL subclass distribution. Lipid stains, such as Sudan Black, may improve the results of HDL electrophoresis after DGU, though further study into the effect of isolation method on HDL cholesterol content should be performed to prevent confounded results.

Proteomic analysis revealed large differences in the protein content of HDL isolated by SDU and DGU. Of the 103 proteins that met the criterion of presence in 50% of samples, 44 proteins were identified only on HDL isolated by DGU. These were predominantly proteins involved in innate immunity, such as immunoglobulins and complement factors typically found in plasma and were therefore likely to be contaminating proteins. Seven proteins were identified only on HDL isolated by SDU. These included CETP and SAA-1, both key HDL proteins involved in its remodelling. This may not necessarily mean that these were not present on HDL from DGU but rather they may be vastly reduced in relative abundance which would not be detected due to the way LFQ intensities are calculated. The remaining 52 proteins were identified on HDL isolated by both techniques, including apolipoproteins and PON-1. Plotting those proteins identified on HDL from both techniques on a qualitative heatmap revealed a distinct pattern of protein abundances for each isolation technique, with apolipoproteins more abundant on HDL from SDU and immune- and coagulation-related proteins more abundant on HDL from DGU. Univariate analysis of apoA-I, apoA-II and PON-1, all well established as HDL proteins, revealed significantly higher abundance of these proteins when isolated by SDU compared to DGU. Taken together, the proteomics findings suggest that isolation by SDU results in cleaner HDL fractions with less contamination by plasma proteins compared to those isolated by DGU. The higher abundance of plasma proteins in DGU may have obscured the detection of strongly associated HDL proteins such as apolipoproteins, given the relative nature of LFQ intensities. These findings also corroborated the higher HDL total protein content after DGU. However, given the wide range of proteins known to associated with HDL (10) it cannot be ruled out that some of the DGU proteins are not contaminants, but loosely associated HDL proteins that are lost in SDU, perhaps due to the ionic strength of the density solution or the longer centrifugation time. There is limited literature directly comparing ultracentrifugation techniques for downstream HDL proteomic analyses. A 2019 meta-analysis of the HDL proteome saw a large degree of heterogeneity of identified proteins by HDL isolation technique, but noted that ultracentrifugation was the most reproducible method (31). Our findings, using the same plasma samples in each method, confirm that there is considerable heterogeneity in the HDL proteome between ultracentrifugation techniques.

Finally, the anti-inflammatory function of HDL isolated by both SDU and DGU was compared. When exposed to HMEC-1 based on 300 μg/mL apoA-I, HDL isolated by DGU was 20 percentage points more effective at reducing TNF*α* stimulated VCAM-1 expression compared to HDL isolated by SDU. This may be partly due to the biologically inert iodixanol having little impact on cellular functions, whereas residual NaBr in the HDL may affect HMEC-1 function. However, the confounding impact of the higher protein content and abundance of immune related proteins on HDL isolated by DGU on the measured anti-inflammatory function cannot be discounted. These proteins can also prevent endothelial inflammation, for example, IgG and its fragments were effective in reducing VCAM-1 expression in response to TNF*α* in primary endothelial cells (32). These findings contrast with the findings of Lemmers et al. (20), who saw no difference in HDL anti-inflammatory function between the two techniques. This may be due to the differences in the ultracentrifugation techniques used compared to the present study; sequential salt density ultracentrifugation was performed in three steps totalling 108 h of centrifugation, while the iodixanol technique employed first removed apoB containing proteins before recovering HDL. This may have had the effect of removing abundant plasma proteins in both instances resulting in cleaner HDL fractions, and removing confounding protein effects, though this was not measured in the study. Due to the large differences in HDL protein content and the potential interference of iodixanol in the cholesterol quantitation assay, HDL anti-inflammatory function was not corrected for HDL protein or cholesterol content. HDL-C was therefore included as a covariate when comparing HDL anti-inflammatory function, to account for individual differences in HDL and protein concentration when dosing based on apoA-I.

These findings also have implications for clinical research settings in which HDL composition and function are being explored as potential biomarkers of cardiometabolic risk. For example, the enrichment of immune- and coagulation-related proteins in DGU-isolated HDL, together with interference in cholesterol and protein quantitation, suggest that DGU may highlight features that resemble disease-associated HDL remodelling. This is important when subtle changes in HDL-associated proteins may reflect HDL dysfunction. The lack of standardised HDL isolation methods and downstream proteomic analysis methods remains a barrier to the clinical utility of HDL metrics beyond HDL-C.

Limitations of this study include the small sample size, which was chosen to preserve samples from the parent study. Long-term frozen storage of plasma modifies the amino acid and lipid metabolome (33, 34), and may shift the distribution of apolipoproteins between lipoprotein classes (35). Other reports found measures of lipid markers including HDL-C remained stable over 13 years within the same sample (36). All of the samples used in this study were stored for the same length of time and all HDL fractions were isolated concurrently so time of storage did not factor into the method comparison. Buffer exchange to remove iodixanol was only performed as a peptide clean-up step before mass spectrometry to protect the instrument, not before each measure of composition and function. Iodixanol may have interfered in the assays despite it being biologically inert. We attempted to measure paraoxonase-1 activity in HDL fractions as a proxy for antioxidant function but the absorbance maximum of iodixanol overlaps with that of the reaction product, precluding the use of the assay. Further validation of the functional differences observed between the isolation methods should be performed using other assays, such as cholesterol efflux capacity or the conjugated diene assay for whole particle antioxidant activity (37).

In conclusion, these findings suggest that for the compositional and anti-inflammatory function study of HDL, SDU results in cleaner HDL fractions with less plasma protein contamination than DGU. Iodixanol appeared to interfere with the quantitation of HDL cholesterol content by colorimetric assay and assessment of HDL subclass distribution which meant that these metrics could not be confidently interpreted. Proteomic analysis revealed that key HDL proteins were of significantly lower abundance in HDL isolated by DGU, which, when coupled with the increase in abundance of proteins known to reduce endothelial activation, introduces potential confounding factors into the interpretation of the anti-inflammatory function assay. It is therefore concluded that further steps to isolate ‘cleaner’ HDL with iodixanol gradients should be employed, but that SDU is better suited to studies of HDL composition and function.

## Data Availability

All data presented in this study can be found in the article or supplementary file. The raw proteomics data can be found at https://doi.org/10.5281/zenodo.19922264.
